# Pseudo-precocious Puberty Associated with an Adrenocortical Tumor in a Young Child

**DOI:** 10.7759/cureus.6440

**Published:** 2019-12-22

**Authors:** Shahd E Kafi, Eid Alagha, Mohamed Abdelmaksoud Shazly, Abdulmoein Al-Agha

**Affiliations:** 1 Pediatrics, King Abdulaziz University Hospital, Jeddah, SAU

**Keywords:** adrenal tumor, young children, pseudoprecocious, puberty, pseudo-precocious

## Abstract

Precocious puberty is commonly observed in pediatric practice due to different causes, including adrenal tumors. Adrenocortical tumors are rare in children and are characterized by an androgenic hormonal excess that causes pseudo-precocious puberty. We present the case of a four-year-old boy with a history of penile enlargement associated with the growth of pubic hair, facial acne, and three-years advanced bone age. Based on hormonal assays, the diagnosis of pseudo-precocious puberty was confirmed. Abdominal magnetic resonance imaging (MRI) revealed a right-sided, retroperitoneal, well-defined adrenal tumor. This case report emphasizes the aim to increase the awareness of adrenocortical tumor as a rare cause of pseudo-precocious puberty in young children.

## Introduction

Although adrenocortical tumors (ACTs) can occur at any age, they are rare in childhood [1]. The first case of childhood ACT was reported in 1865 [2]. The ACT is a rare neoplasm that accounts for about 0.2% of all tumors affecting children and approximately 6% of all adrenal tumors observed in the pediatric group [3]. The annual incidence of ACT in children under 15 years of age is extremely rare, ranging from 0.3-0.5 cases per million [4]. However, the incidence varies across various geographical regions; for example, in the United States and Europe, it accounts for 0.3-0.38 cases per million [4-5]. In comparison, 10-15 times more cases are diagnosed in Brazil than in the rest of the world [6-7]. Almost 50% of ACT cases are associated with syndromes, and the two main syndromes that were reported included the Li-Fraumeni syndrome that resulted from alterations of the tumor suppressor gene p53 in chromosome 17 and the Beckwith-Weidman syndrome that resulted from the effect of the region located in chromosome 11p15 [2,8-9]. ACT was categorized as functional (hormone-secreting), which is most commonly found in children and adolescents, or non-functional (silent), which is usually found in adults with symptoms of abdominal discomfort or back pain caused by the large mass of the tumor [10-11]. ACT can be benign or malignant [3]. The most common clinical presentation of ACT in children is pseudo-precocious puberty, which is observed in approximately 50%-84.2% of cases or as Cushing’s syndrome in the remaining cases [12-13]. However, malignancy should not be underestimated in this age group [2]. We report the case of a four-year-old boy who presented with pseudo-precocious puberty caused by ACT, which is rare in children.

## Case presentation

**Figure 1:**
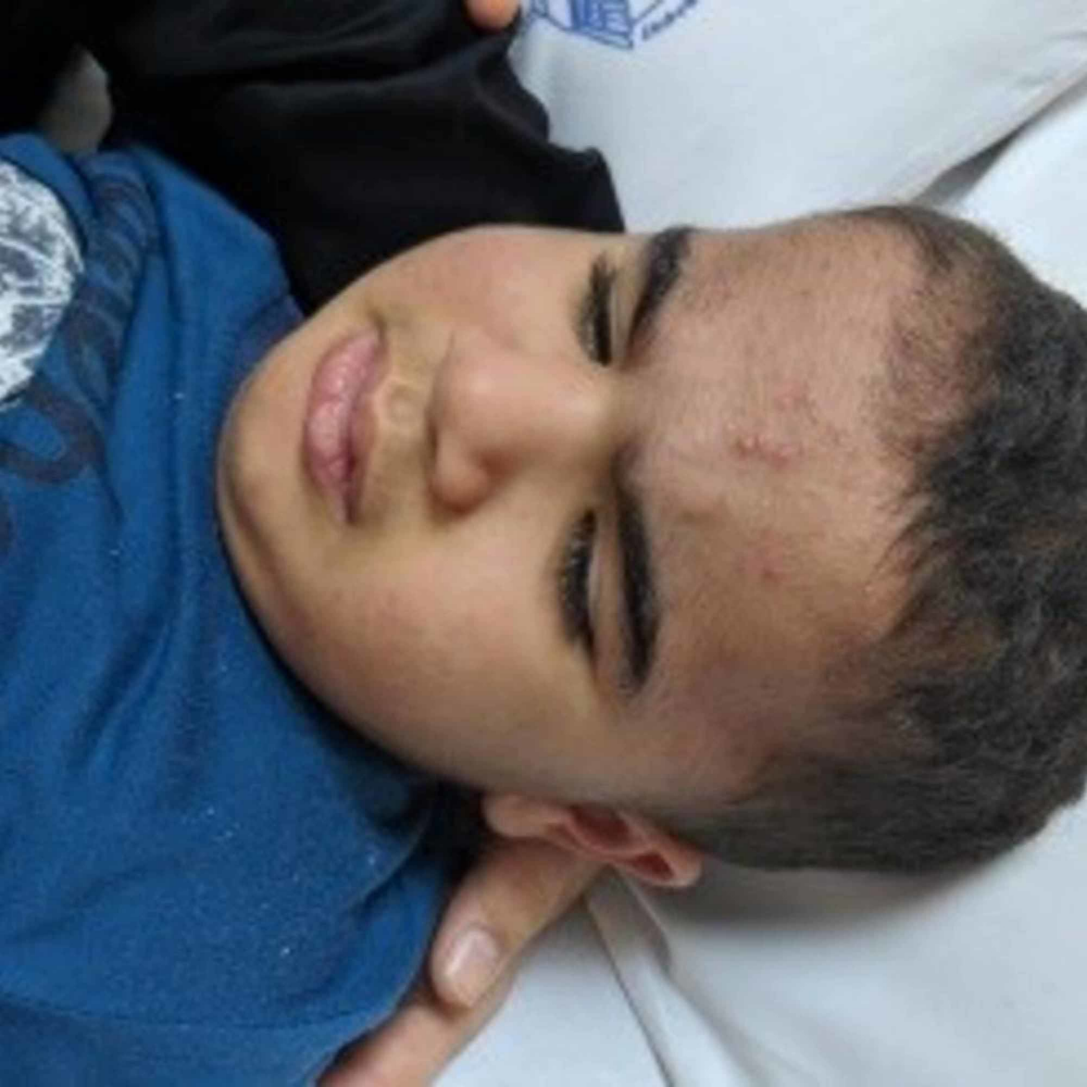
Facial acne was observed as a part of pubertal changes

**Figure 2:**
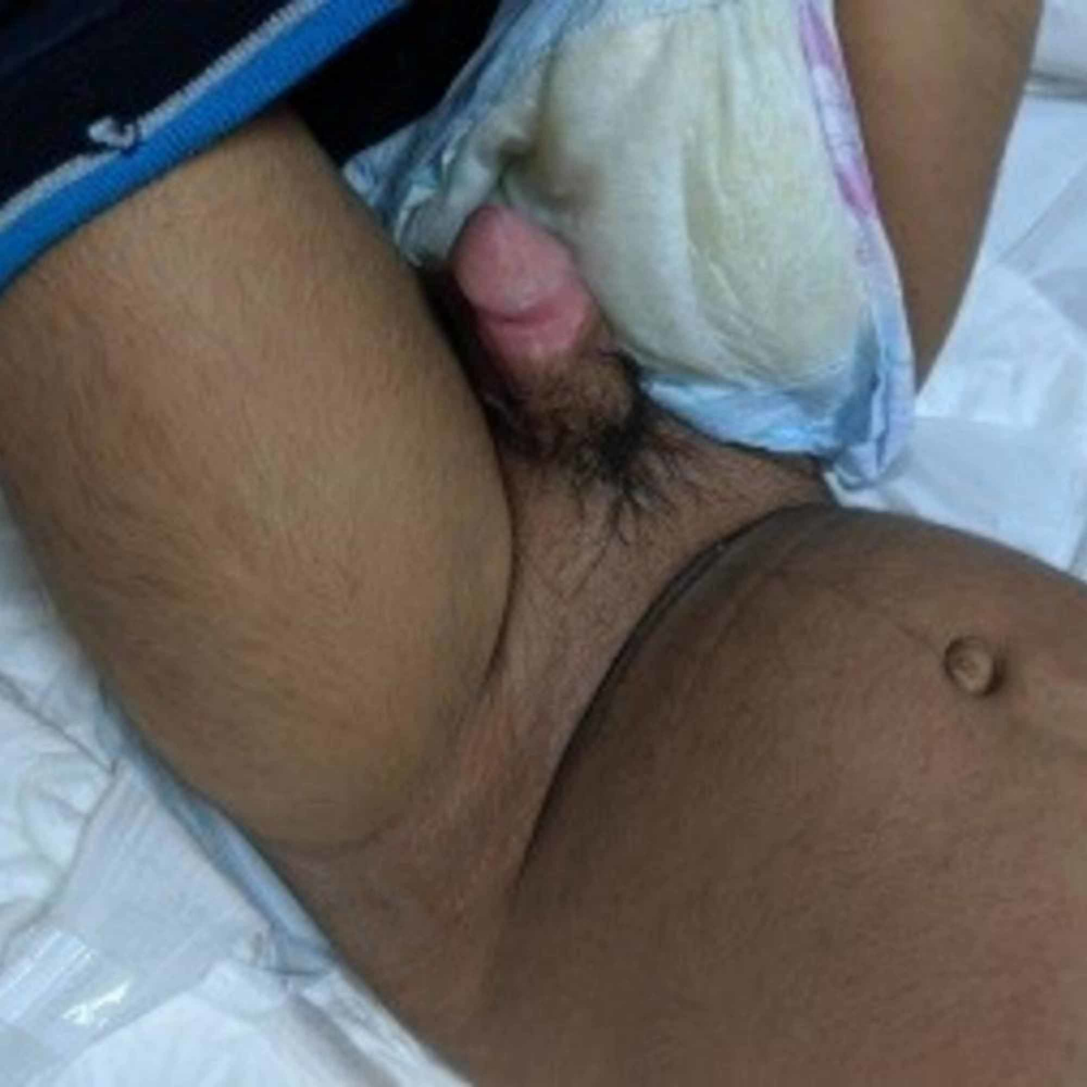
Patient with erected penis with increase in penile width, length, and pubic hair

**Figure 3:**
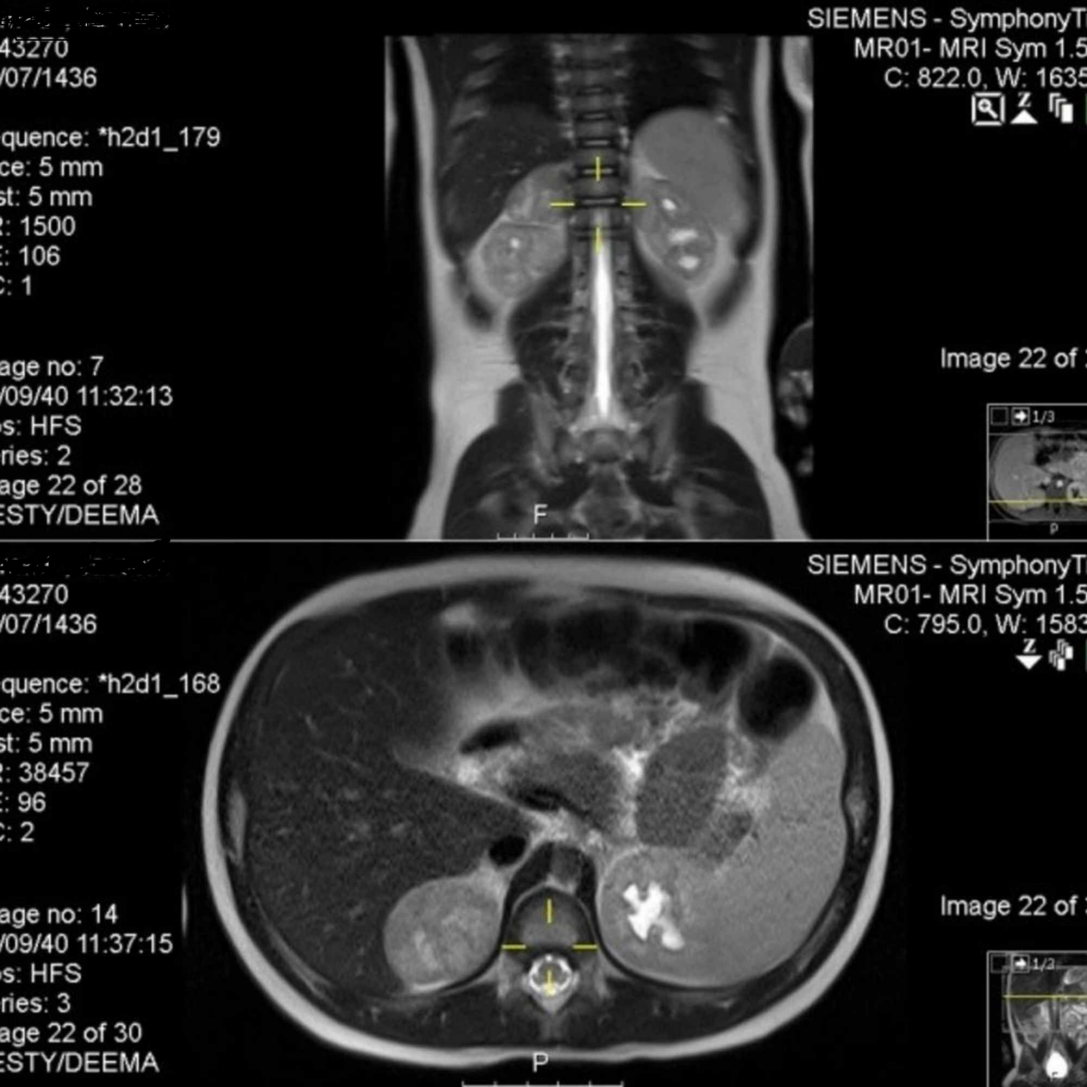
Magnetic resonance imaging showing an adrenal tumor

A four-year-old boy presented to the pediatric endocrinology clinic with the appearance of pubic hair in conjunction with an increase in penile length for two months. He was delivered vaginally after a full-term pregnancy and had no medical or surgical histories. There was no family history of endocrine tumors or early puberty. Systemic examinations, including vital signs, were unremarkable. He had facial acne on his forehead and cheeks (Figure 1) with no skin hyperpigmentation. His Tanner staging of pubic hair and genitalia was stage III. The volume of both the testes was 2 ml, and his stretched penile length (SPL) was 7.5 cm (Figure 2). His serum hormonal assays are presented in Table 1 and show low basal levels of follicle-stimulating hormone (FSH), luteinizing hormone (LH), and 17-hydroxyprogesterone and elevated levels of testosterone and dehydroepiandrosterone sulfate (DHEA-S). Gonadotropin-releasing hormone (GnRH) stimulation test showed a negative response. The bone age was advanced by three years. Therefore, the diagnosis of pseudo-precocious puberty was confirmed. Abdominal magnetic resonance imaging (MRI) revealed a right-sided retroperitoneal adrenal mass (Figure 3). The adrenocortical tumor was diagnosed based on clinical manifestations and imaging.

**Table 1:** Results of the hormonal assay 17-hydroxyprogesterone (17-OH progesterone); alfa fetoprotein (AFP); beta human chorionic gonadotropin (BHCG)

Test	Date	Result
Follicular stimulating hormone	0 min	0.2
	15 min	0.4
	30 min	0.3
	45 min	0.4
	60 min	0.3
	90 min	0.5
	120 min	0.6
Luteinizing hormone	0 min	0.2
	15 min	0.3
	30 min	0.2
	45 min	0.2
	60 min	0.2
	90 min	0.2
	120 min	0.2
Testosterone	first	39
	repeated	33
17 OH progesterone	18.8 ng/ml	Normal
Alfa fetoprotein (AFP)	4.45 ng/ml	normal
Beta-human chorionic gonadotropin (BHCG)	<1.2 iu/ml	normal

## Discussion

Precocious puberty is defined as the presence of secondary sex characteristics before the age of eight in girls and nine in boys. These limits represent 2-2.5 standard deviations (SD) below the mean age of onset of puberty [14]. Precocious puberty is of two main types: central precocious puberty (CPP), which is gonadotropin dependent and caused by the early activation of the hypothalamic-pituitary-gonadal axis, and pseudo-precocious puberty, which is gonadotropin independent and results from the excess secretion of sex hormones from the gonads or adrenal glands [15]. There are several causes of pseudo-precocious puberty, including adrenal tumors, non-classical congenital adrenal hyperplasia, testicular tumors, germ cell tumors, McCune-Albright syndrome, familial male-limited precocious puberty (testotoxicosis), or exogenous sex steroid hormone usage. The clinical presentations of the two types of precocious puberty are markedly different, as CPP often presents with gonadal enlargement of testicles in males or ovaries in females, whereas pseudo-precocious puberty presents mainly with the early development of pubic and/or axillary hair. The dynamic stimulating test of the hypothalamic-pituitary-gonadal axis using a gonadotropin-releasing hormone (GnRH) agonist has an essential and golden role in the differentiation of the two types of precocious puberty. Pubertal response by increasing the hormonal level of LH and FSH is usually observed in CPP, while no response usually confirms pseudo-precocious puberty, as observed in this case.

Adrenocortical tumors (ACTs) can cause pseudo-precocious puberty by the secretion of excess androgenic hormones leading to virilization, which is the most common symptom of tumors in childhood. However, a late presentation of ACT could be seen with CPP secondary to the maturation of the hypothalamic-pituitary-gonadal axis. Female children are more affected than males, with a peak age of younger than four years [12]. The management of this disease is still challenging because of the limited number of reported cases. Nonclassical congenital adrenal hyperplasia (NCAH) causes androgen oversecretion, which leads to pseudo-precocious puberty with the development of early signs of puberty, including pubic hair, acne, and accelerating skeletal maturation [16]. This disease can be detected early in the neonatal period by screening for an elevated serum level of 17-hydroxyprogesterone. However, in this case, NCAH was excluded because of normal levels of 17-hydroxyprogesterone, cortisol, and adrenocorticotropic hormone (ACTH). An abdominal MRI finding of unilateral tumor of the right adrenal gland confirms the diagnosis of ACT.

A literature review of case reports revealed that virilization was considered a good clue for suspecting ACT, similar to the presentation in this case [11-13]. Razavi reported a 10-month-old boy diagnosed with ACT, who presented with sexual pubic hair, acne, and seborrhea dermatitis [3]. Another case of a two-year-old Indian boy presented with pubic hair and penile enlargement was reported [1]. Ghazizadeh reported virilization in a two-year-old girl associated with ACT in 2013 [16]. However, discriminating benign tumors from malignant tumors does not depend on hormone secretions. A comprehensive evaluation of precocious puberty, including clinical manifestations, laboratory testing, and imaging, is important for reaching the final diagnosis.

## Conclusions

Virilization of both sexes is an important manifestation of adrenocortical tumors, which is one of the rare causes of pseudo-precocious puberty. A high index of suspicion and an increased awareness of pediatricians play an important role in the early diagnosis and treatment of this disease.
